# More Severe Insomnia Complaints in People with Stronger Long-Range Temporal Correlations in Wake Resting-State EEG

**DOI:** 10.3389/fphys.2016.00576

**Published:** 2016-11-29

**Authors:** Michele A. Colombo, Yishul Wei, Jennifer R. Ramautar, Klaus Linkenkaer-Hansen, Enzo Tagliazucchi, Eus J. W. Van Someren

**Affiliations:** ^1^Department of Sleep and Cognition, Netherlands Institute for Neuroscience, An Institute of the Royal Netherlands Academy of Arts and SciencesAmsterdam, Netherlands; ^2^Bernstein Center Freiburg and Faculty of Biology, University of FreiburgFreiburg, Germany; ^3^Centre for Chronobiology, Psychiatric Hospital of the University of Basel (UPK)Basel, Switzerland; ^4^Department of Integrative Neurophysiology, Center for Neurogenomics and Cognitive Research, Vrije Universiteit AmsterdamAmsterdam, Netherlands; ^5^Department of Psychiatry/GGZ inGeest, VU University Medical CenterAmsterdam, Netherlands

**Keywords:** resting-state, insomnia, sleep, HD-EEG, long-range temporal correlations, criticality, detrended fluctuation analysis, excitation-inhibition balance

## Abstract

The complaints of people suffering from Insomnia Disorder (ID) concern both sleep and daytime functioning. However, little is known about wake brain temporal dynamics in people with ID. We therefore assessed possible alterations in Long-Range Temporal Correlations (LRTC) in the amplitude fluctuations of band-filtered oscillations in electroencephalography (EEG) recordings. We investigated whether LRTC differ between cases with ID and matched controls. Within both groups, we moreover investigated whether individual differences in subjective insomnia complaints are associated with LRTC. Resting-state high-density EEG (256-channel) was recorded in 52 participants with ID and 43 age- and sex-matched controls, during Eyes Open (EO) and Eyes Closed (EC). Detrended fluctuation analysis was applied to the amplitude envelope of band-filtered EEG oscillations (theta, alpha, sigma, beta-1, beta-2) to obtain the Hurst exponents (*H*), as measures of LRTC. Participants rated their subjective insomnia complaints using the Insomnia Severity Index (ISI). Through general linear models, we evaluated whether *H*, aggregated across electrodes and frequencies, differed between cases and controls, or showed within-group associations with individual differences in ISI. Additionally, we characterized the spatio-spectral profiles of group differences and associations using non-parametric statistics. *H* did not differ between cases with ID and controls in any of the frequency bands, neither during EO nor EC. During EO, however, within-group associations between *H* and ISI indicated that individuals who experienced worse sleep quality had stronger LRTC. Spatio-spectral profiles indicated that the associations held most prominently for the amplitude fluctuations of parietal theta oscillations within the ID group, and of centro-frontal beta-1 oscillations in controls. While people suffering from insomnia experience substantially worse sleep quality than controls, their brain dynamics express similar strength of LRTC. In each group, however, individuals experiencing worse sleep quality tend to have stronger LRTC during eyes open wakefulness, in a spatio-spectral range specific for each group. Taken together, the findings indicate that subjective insomnia complaints involve distinct dynamical processes in people with ID and controls. The findings are in agreement with recent reports on decreasing LRTC with sleep depth, and with the hypothesis that sleep balances brain excitability.

## Introduction

Complaints of insomnia are estimated to affect up to a third of the general population (Ohayon, 2002) and constitute the key connecting symptom in the network of associations between psychopathological symptoms (Borsboom et al., 2011). Insomnia complaints concern perceived problems of sleeping at the beginning, middle or end of the sleep period, as well as their perceived repercussions during daytime. Commonly reported daytime repercussions include fatigue, incapacity to concentrate, altered mood, worry, and other people noticing one's sleep problems (Bastien et al., 2001). These insomnia complaints are mostly transient, but if they recur at least three times per week for more than 3 months, Insomnia Disorder may be diagnosed (American Psychiatric Association, 2013).

Insomnia Disorder is characterized by chronic hyperarousal that can be found across cognitive, emotional, somatic and neurobiological domains (Bonnet and Arand, 1997; Riemann et al., 2010). Multiple neurobiological pathways could underlie hyperarousal in Insomnia Disorder, including an imbalance in the activity of wake and sleep promoting nuclei (Cano et al., 2008) and of networks regulating emotion, reward and cortical excitability (Altena et al., 2010; Stoffers et al., 2014; Wassing et al., 2016). It is hypothesized that hyperarousal involves elevated cortical excitability, resulting from attenuated inhibitory and heightened excitatory processes in neuronal networks (Van der Werf et al., 2010). This imbalance manifests as a shift in power from lower to higher frequency oscillations in resting state electroencephalography (EEG) (Wolynczyk-Gmaj and Szelenberger, 2011; Corsi-Cabrera et al., 2012; Colombo et al., 2016). It moreover manifests as reduced gating and heightened sensory reactivity in response to exogenous (Yang and Lo, 2007; Bastien et al., 2008; Hairston et al., 2010; Kertesz and Cote, 2011) and endogenous stimuli (Wei et al., 2016).

Recently, complex dynamic theory has been used to describe the process of sleep (Lo et al., 2002, 2004, 2013). Sleep is hypothesized to regulate the complex organization of brain dynamics (Pearlmutter and Houghton, 2009), by keeping excitatory and inhibitory processes balanced (Huber et al., 2013). While prolonged wakefulness increases brain excitability, sleep reduces it, preventing an imbalance towards excitation that would favor runaway seizure-like activity (Meisel et al., 2013, 2015). Therefore, important pathophysiological mechanisms underlying insomnia complaints may be unveiled by studying the complex organization of brain dynamics.

The dynamics of brain activity show a complex spatio-temporal organization that is autocorrelated over multiple scales. Accordingly, local short-lived activity can trigger far-reaching consequences over space and time (Hesse and Gross, 2014). In particular, the temporal organization of brain dynamics can be characterized by their Long-Range Temporal Correlations (LRTC): autocorrelations that decay over time according to a power law (Chialvo, 2010; Poil et al., 2012; Tagliazucchi et al., 2012). LRTC of brain dynamics reflect a memory of the system that can span tens and even up to several hundreds of seconds (Linkenkaer-Hansen et al., 2001; Kantelhardt et al., 2015). LRTC of brain dynamics have been observed with functional magnetic resonance imaging (Tagliazucchi et al., 2012), magnetoencephalography (Linkenkaer-Hansen et al., 2001), EEG (Hardstone et al., 2012), and stereotactic EEG (Zhigalov et al., 2015).

**Figure 1:**
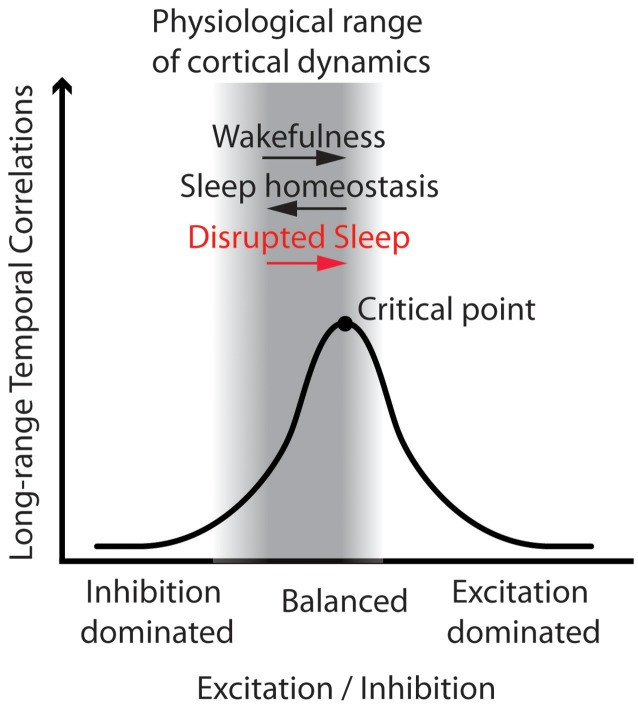
**Working model: Disrupted sleep involves higher excitation to inhibition ratio, and thus stronger Long-Range Temporal Correlations (LRTC) in wake brain dynamics**. The figure illustrates that LRTC peak at the critical point, when excitation and inhibition of a neuronal network are balanced. In the physiological range of brain dynamics—near and below the critical point—an increase in the excitation to inhibition ratio entails stronger LRTC. Here we put forth the hypothesis that disrupted sleep would increase the excitation to inhibition ratio, and thus increase LRTC. We therefore expected stronger LRTC in people with Insomnia Disorder as compared to matched controls, and that, in each group, the individuals with more severe insomnia complaints would also show stronger LRTC. This hypothetical model is general and does not make specific predictions for each frequency band and electrode, where LRTC can be measured. We therefore investigated the model predictions on a global aggregated measure of LRTC, and then we explored associations at a refined spatio-spectral level.

Computational models have shown that LRTC emerge when excitatory and inhibitory processes of a neuronal network are balanced, near a critical transition between order and disorder (Poil et al., 2012). LRTC reach a maximum at the critical point and decay with the distance from it. Below the critical point the network is dominated by inhibition, whereas above it, by excitation (Poil et al., 2012). Convergent evidence based on several species and recording techniques as well as computer models (Priesemann et al., 2014) confirms the hypothesis that physiological brain dynamics are typically poised near and below the critical point (Pearlmutter and Houghton, 2009; Carhart-Harris et al., 2014). Under this hypothesis, stronger LRTC are therefore indicative of a higher excitation to inhibition ratio (Poil et al., 2012) (see Figure 1).

The aim of the present study was to assess whether LRTC in the amplitude fluctuations of band-filtered EEG oscillations, which are affected by the balance between excitation and inhibition, are more persistent (1) in people suffering from Insomnia Disorder as compared to matched controls, and (2) in association with the subjective severity of insomnia complaints within each group. For this purpose, we recorded high-density EEG (HD-EEG) in people with Insomnia Disorder and controls, during Eyes Open (EO) and Eyes Closed (EC) resting-state conditions, and quantified LRTC using Hurst exponents obtained from detrended fluctuation analysis (DFA) of the amplitude envelope of several band-filtered oscillations (Kantelhardt et al., 2001; Hardstone et al., 2012). This allowed us to explore group and individual differences in LRTC at a fine-grained spatio-spectral level, next to testing hypothesis on a global aggregated measure of LRTC. We hypothesized that LRTC would be globally elevated in people with Insomnia Disorder as compared to matched controls, and that LRTC would globally positively correlate with the severity of insomnia complaints (Figure 1). Furthermore, in order to evaluate whether the severity of insomnia complaints is associated with similar or distinct brain dynamical processes in people with Insomnia Disorder and controls, we explored whether the correlations have different spatio-spectral profiles for the two groups.

## Methods

### Participants

Participants were recruited through advertisements and the Netherlands Sleep Registry (Benjamins et al., 2013). Telephone screening and subsequent face-to-face interview were conducted to exclude potential participants: with any psychiatric or neurological illness; with a history of sleep apnea, restless leg syndrome, narcolepsy, circadian disorders or chronic sleep deprivation; who have used hypnotics in the previous 2 months. The criteria for the Insomnia Disorder (ID) group adhered to the DSM-5 diagnosis (American Psychiatric Association, 2013), complemented by an Insomnia Severity Index (ISI) score equal or larger than the sub-clinical cutoff of 8 (Bastien et al., 2001). The controls (CTRL) group, age- and sex-matched to the ID group (Supplementary Material), reported neither severe nor persistent insomnia complaints and had an ISI score smaller than 8. The ISI is the sum-score of seven Likert-scale items (graded on five levels of agreement) concerning insomnia complaints, including sleep problems and their perceived impact on wakefulness, within the past 2 weeks (Bastien et al., 2001; Morin et al., 2011). The ISI was thus used as an index of the severity of insomnia complaints. We included 52 participants with ID (43 females), aged (range, mean ± standard deviation) 21–69, 50.23 ± 13.31 year, and 43 CTRL (32 females), aged 22–70, 46.1 ± 14.9 year. Participants signed informed consent; the study was approved by the ethical committee of the VU University Medical Center, Amsterdam, The Netherlands.

### Recordings

Participants were instructed to maintain a regular sleep/wake schedule during the 2 weeks prior to laboratory assessment. Moreover, on the day of laboratory assessment, they were also instructed to refrain from alcohol and drugs and to limit their intake of caffeinated beverages to a maximum of two cups, which were allowed only before 12:00 pm. EEG was recorded between 19:15 and 23:45 pm. During the recordings, participants were instructed not to move their head and not to fall asleep while seated in an upright position in two wake resting-state conditions: 5 min of visual fixation on a cross hair on a monitor (Eyes Open, EO), followed by 5 min with Eyes Closed (EC). High-density EEG (HD-EEG) was recorded using a 256-channel system, connected to a Net Amps 300 amplifier (Electrical Geodesic Inc., Eugene, OR, input impedance: 200 MΩ, A/D converter: 24 bits). Electrode impedance was kept below 100 kΩ. Signals were acquired with a sampling rate of 1000 Hz and with a Cz reference.

### Preprocessing

All preprocessing steps were coded in MATLAB (The Mathworks Inc., Natick, MA; version 8.3), using the MEEGPIPE toolbox (https://github.com/meegpipe/meegpipe). Large non-physiological deviations with non-stereotypical time-course were removed after estimation by local polynomial approximation through the LPA-ICI algorithm (Katkovnik et al., 2006). Signals were subsequently downsampled to 250 Hz with an antialiasing filter, and then band-pass filtered using a Hamming-windowed sinc Type I digital FIR filter (Widmann and Schröger, 2012) (cutoffs: 0.75–65 Hz; transition bandwidth: 0.2 and 5 Hz respectively for each end). Electrodes, first, and epochs, later, were evaluated for rejection using two similar automated procedures, adaptive to each EEG recording (Colombo et al., 2016). Further artifacts from physiological (cardiac field, eye movements/blinks, muscle tension) and non-physiological (power-line and sparse-sensor noise) sources were removed using automated procedures. Electrodes located on the neck and the face were excluded from further analysis; the remaining 183 scalp electrodes were re-referenced to the common average. LRTC were estimated over the first 3 min of the cleaned data.

### Estimation of LRTC

**Figure 2:**
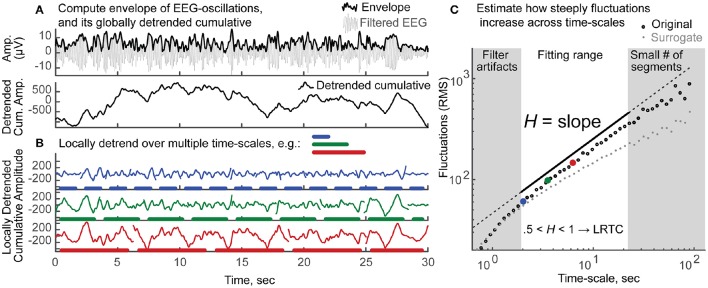
**Schematic explanation of the estimation of ***H***, the Long-Range Temporal Correlations scaling exponent of the amplitude envelope, through detrended fluctuation analysis (DFA). (A)** Top: The amplitude envelope (*thick black line*) is the absolute value of the Hilbert transform of the band-pass filtered oscillation (*thin gray line*). Only 30 s out of 3 min of alpha band oscillation are shown for clarity. Bottom: The cumulative sum of the envelope in top is shown after subtraction of its global linear trend. **(B)** This cumulative sum time series is divided into non-overlapping segments of different sizes (e.g., *blue*, 2.02 s; *green*, 3.55 s; *red*, 6.55 s); local third-order polynomial trends are removed. The residual signals display a clear increase in fluctuations, as the time-scale increases (blue < green < red). **(C)** This increase in fluctuations—measured as the average root mean square (RMS) of the detrended segments—as a function of the time-scale of reference, can be quantified by a power-law. Twenty logarithmically-spaced time-scales are considered to fit a least-squares line over log-log axes. The fitting range is chosen so that filter artifacts are minimal (>8 times the filter order), yet enough segments are available for a reliable estimate of the fluctuation (>8 segments). The scaling exponent of the amplitude envelope *H* is the slope of the fitted line (in black, shifted vertically for clarity). Values obtained from the original signal are shown in black circles; large dots in blue, green and red correspond to the time-scales exemplified in **(B)**. The average values of 100 surrogate signals (each being the envelope of band-pass filtered white noise, rescaled to the mean amplitude of the original signal) are shown in gray, for visual comparison. The original signal, as compared to the surrogate, shows a steeper increase in fluctuations across time-scales, reflecting that EEG dynamics are more strongly autocorrelated than those of a random process. *H* between 0.5 and 1 indicates the presence of positive autocorrelations. These dependencies persist beyond the time-scale of the oscillations (10^−2^ – 10^−1^) up to tens of seconds, and are thus named Long-Range Temporal Correlations (LRTC).

In order to quantify LRTC, we applied Detrended Fluctuation Analysis (DFA) (Kantelhardt et al., 2001; Hardstone et al., 2012) to the amplitude envelope of the band-pass filtered EEG signals, so as to estimate the corresponding Hurst scaling exponent *H*, for each frequency band and for each electrode. While the preprocessing procedure removed large artifactual periods and corrected for various sources of noise, we do not exclude that the data may still be contaminated by minor artifacts. Even in this case—where the signals are short, have portions of the data cut out and are partially contaminated by artifacts—the estimation of the Hurst exponents through DFA is reliable (Chen et al., 2002; Ma et al., 2010). EEG signals were filtered in the frequency bands of theta (4–8 Hz), alpha (8–12 Hz), sigma (12–15 Hz), beta-1 (15–22), beta-2 (22–30 Hz), using Hamming-windowed sinc FIR filters with window sizes of 125, 63, 38, 31, 23 data points, respectively (Widmann and Schröger, 2012). The amplitude envelope was then obtained as the absolute value of the Hilbert transform (Figure 2A, top). The globally detrended cumulative envelope time-series was obtained by cumulatively summing the envelope over the duration of the recording, and removing its global linear trend (Hardstone et al., 2012) (Figure 2A, bottom). This time-series was split into non-overlapping segments, from which local third-order polynomial trends were estimated with least squares (following Kantelhardt et al., 2015) and subtracted (Figure 2B). The fluctuation was quantified as the average root mean square (RMS) of all locally-detrended segments. The process was repeated for segments of different time-scales: 20 logarithmically-spaced time-scales were used between a minimum, eight times larger than the filter order (for theta to beta-2, respectively: 4, 2.02, 1.24, 1, 0.74 s), and a maximum, eight times smaller than recording length (22.5 s). Note that we did not consider smaller time-scales so as to avoid biasing the scaling-law estimation from short-range autocorrelations induced by the temporal filter (Hardstone et al., 2012). Subsequently, plotting the average RMS vs. time-scale on a log-log scale produced a nearly linear sequence of values (Figure 2C). The Hurst scaling exponent of the amplitude envelope, *H*, is the slope of the least-squares linear fit. Thus, *H* quantifies how steeply the fluctuations increase with the time-scale of reference. *H* between 0 and 0.5 indicates negative autocorrelations; *H* equal to 0.5 indicates no autocorrelation (random process); *H* between 0.5 and 1 indicates positive autocorrelations (LRTC); and *H* above 1 indicates the process is non-stationary. Consistently with the existing literature on neurophysiology during wakefulness, we use the term LRTC to refer to positive autocorrelations—estimated with *H—*that persist up to tens of seconds (e.g., Linkenkaer-Hansen et al., 2001; Bornas et al., 2013; Palva et al., 2013). However, the reader should notice that in the context of long sleep recordings, a fitting range of 2–20 s is considered short (Kantelhardt et al., 2015).

### *H* group difference and association with subjective insomnia complaints

An aggregated measure of the LRTC scaling exponents was obtained, separately for EO and EC, as the grand-median of the *H* exponents across frequency bands and electrodes. Separately for each of the two resting-state conditions (EO, EC), type-II univariate general linear model (GLM) was used to estimate whether the grand-median *H* differed between groups and was associated with individual differences in the subjective severity of insomnia complaints (measured by the ISI score, range 0–25). The GLM analysis was performed in R (version 3.0.2), with the CAR package (Fox and Weisberg, 2010).

In order to clarify whether the association between the grand-median *H* and ISI held stronger within each group or across groups, we compared the Spearman correlation coefficients obtained within each group and across groups (Supplementary Material).

During either EO or EC, if the GLM indicated the grand-median *H* differed significantly between groups or was significantly associated with the ISI score, follow-up non-parametric tests were performed: Wilcoxon rank-sum tests were used to quantify group differences, Spearman correlations to quantify associations.

### Group-specific spectral and spatio-spectral profiles of correlations between *H* and ISI

For the resting state condition (Eyes Open or Closed) where the ISI was found to be associated with the grand-median *H*, we further investigated the group-specific spectral and spatio-spectral profiles of the association.

First, we determined whether the association between LRTC and the severity of insomnia complaints had a spectral profile specific to each group. For each frequency band, we computed the median *H* across electrodes. We then obtained the Spearman correlation coefficient (*rho*) between ISI and the median *H* exponent in each frequency band, together with the bootstrap confidence intervals (1000 iterations) of the correlation coefficients.

Second, we determined whether the association between LRTC and the severity of insomnia complaints had a spatio-spectral profile specific to each group. A Spearman correlation coefficient and its corresponding *t*-statistic (*df* = 50 and 41, respectively for ID and CTRL) were calculated separately for each spatio-spectral bin (i.e., for each electrode and frequency band). Following the threshold-free cluster enhancement (TFCE) procedure (Mensen and Khatami, 2013), each *t*-statistic was enhanced according to the intensity of the adjacent spatio-spectral bins. The parameters *e* and *h*, corresponding to the exponents of extension and height, were set to the default values, 0.66 and 2 respectively, as derived from random field theory (Mensen and Khatami, 2013). Significance (set at *p* = 0.05) of each enhanced statistic (*t*_*tfce*_) was determined by comparing it to the respective empirical null hypothesis distribution, constructed by Monte Carlo permutation with 1000 iterations. TFCE retains local maxima of the topology of statistical contrasts and avoids the use of arbitrary thresholds to form clusters. Importantly, the TFCE procedure has larger power compared to the common cluster-based inference, while still accounting for multiple comparisons (Mensen and Khatami, 2013).

## Results

### ID and CTRL have similar LRTC

Across all participants, the Hurst scaling exponents *H* of the amplitude envelope fell within the 0.5–1 range, confirming the presence of LRTC in band-filtered EEG amplitude fluctuations (Kantelhardt et al., 2001; Hardstone et al., 2012). The *H* exponent was larger during EO than during EC, significantly so for alpha, sigma, beta-1 and beta-2, and only at trend-level for theta (Supplementary Figure S1A). Between-participants variation of *H* was significantly larger across electrodes during EO than during EC, for alpha, sigma, beta-1 and beta-2 (Supplementary Figure S1B). During EO, *H* was similar for the ID and CTRL groups for all frequency bands (**Figure 4A**) and electrodes (**Figure 4B**). Accordingly, group differences in *H* were not statistically significant, as reported in the following section. Both groups displayed largest *H* exponents in alpha oscillations; they also displayed largest *H* over parietal regions, consistently across frequency bands. Similar results were obtained during EC (Supplementary Figure S1A, topographies not shown).

### During EO, the grand-median hurst exponent increases with ISI, in ID and in CTRL

Univariate GLMs, separately for EC and EO, quantified whether the LRTC scaling exponent *H*, aggregated over frequencies and electrodes, differed between groups and was associated with the severity of insomnia complaints.

During EC, the GLM indicated that the grand-median *H* did not differ between ID and CTRL [*F*_(1, 89)_ = 0.537, *p* = 0.466] and did not change with ISI [*F*_(1, 89)_ = 1.267, *p* = 0.263]. There was also no ID-by-ISI interaction effect [*F*_(1, 89)_ = 1.000, *p* = 0.320].

**Figure 3:**
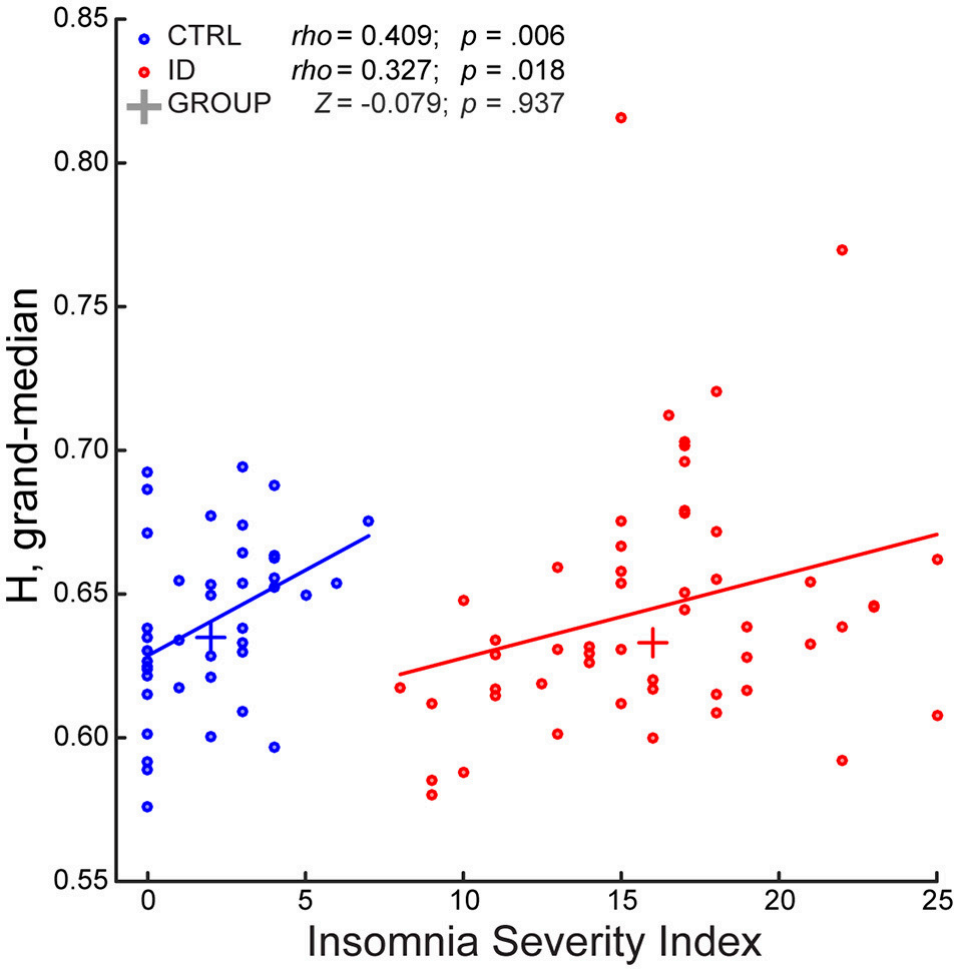
**The Long-Range Temporal Correlations scaling exponent—aggregated across frequencies and electrodes—of EEG amplitude fluctuations during Eyes Open increases with severity of insomnia complaints, in participants with Insomnia Disorder and in matched controls**. The Insomnia Severity Index (ISI) positively correlated with the grand-median of the *H* exponents across frequencies and electrodes, in participants with Insomnia Disorder (ID, red) and in matched controls (CTRL, blue). The groups did not significantly differ with respect to grand-median *H*. A least-squares line is shown for each group; a cross indicates the median point of each group with respect to the two axes. Within-group Spearman correlation coefficients (*rho*), rank-sum *Z*-statistic of group difference in grand-median *H*, and their respective *p*-values are shown on top.

During EO, the GLM indicated a significant effect of group [*F*_(1, 89)_ = 5.397, *p* = 0.022]. However, a follow up rank-sum test revealed that ID did not differ from CTRL with respect to the grand-median *H* (*z* = −0.079; *p* = 0.937). Further investigation of group differences during EO at the fine-grained spatio-spectral level (Supplementary Material) indicated no group differences at any frequency or electrode. Furthermore, the GLM indicated a significant effect of ISI [*F*_(1, 89)_ = 8.397, *p* = 0.005]. Follow-up correlation tests revealed that the grand-median *H* increased with ISI (Figure 3), in ID (*rho* = 0.327, *p* = 0.018) and in CTRL (*rho* = 0.409, *p* = 0.006), while only showing a trend when considering all participants together (*rho* = 0.174, *p* = 0.092) (Supplementary Figure S2). Finally, the GLM indicated no ID-by-ISI interaction effect [*F*_(1, 89)_ = 1.000, *p* = 0.320].

In sum, the groups did not differ with respect to *H*, at any frequency and electrode, either during EO or EC. However, within each group, ISI positively correlated with *H*, aggregated over frequencies, and electrodes, during EO. In the remainder of the Results we accordingly focus on EO, to detail the association between ISI and *H* at a fine-grained level.

### Group-specific spectral and spatio-spectral profiles of correlations between ISI and *H* during EO

**Figure 4:**
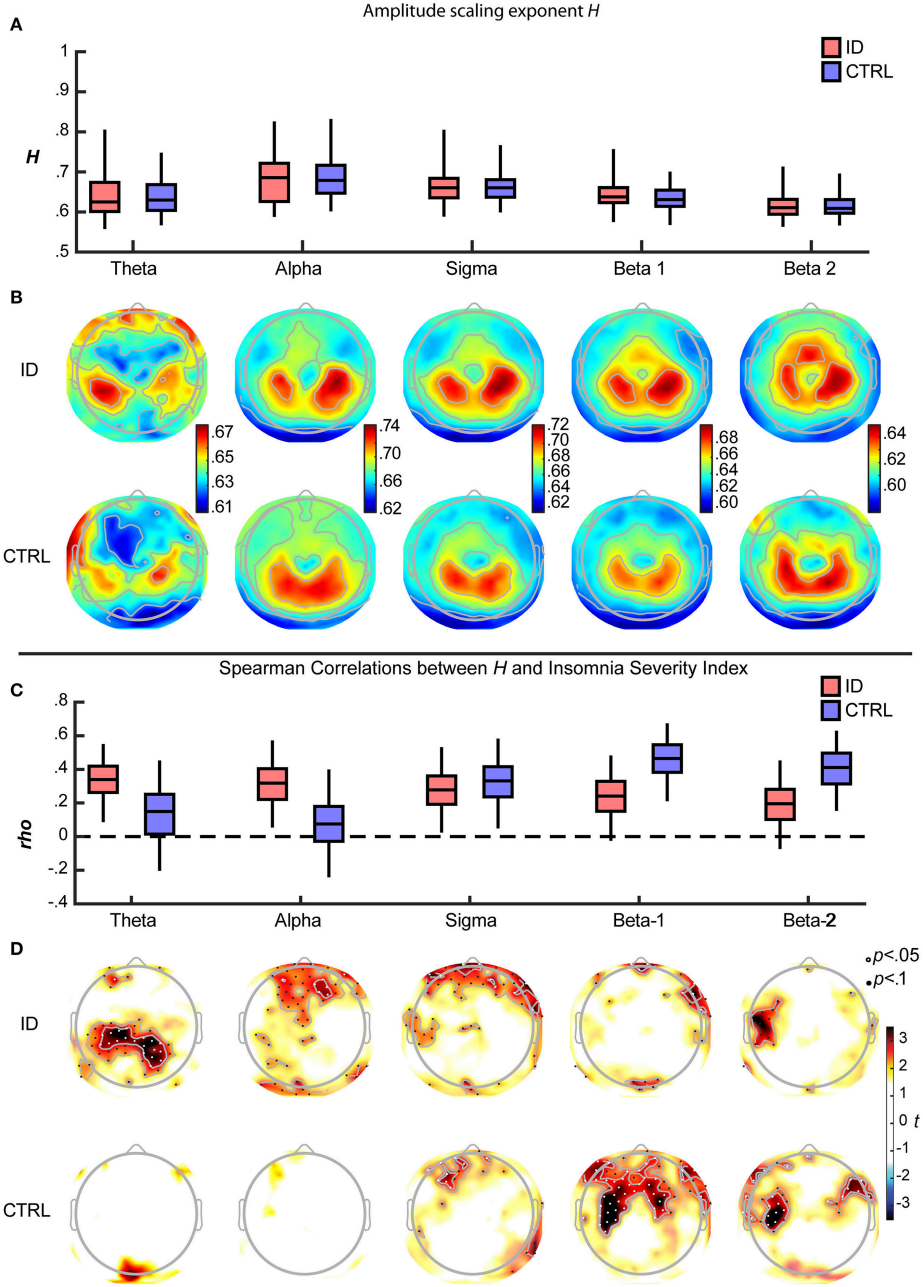
**Long-Range Temporal Correlations in EEG amplitude fluctuations during Eyes Open increase with severity of insomnia complaints, with distinct spatio-spectral profiles in participants with Insomnia Disorder and matched controls. (A)** For each frequency band and group, the *H* exponent (median across electrodes) is displayed, as the median across participants (*middle line*) with inter quartile range (*box*) and the central 95% of participants in each group (*whiskers*). Data are shown for Insomnia Disorder (ID, red) and controls (CTRL, blue) **(B)** Grand average topographies of the *H* exponents are shown for each frequency band and group. **(C)** The *H* exponent (median across electrodes) was positively associated with the Insomnia Severity Index (ISI) in both groups. Boxplots show the bootstrap-distributions of Spearman's correlation coefficients (*rho*). **(D)** Topographies of *t*-values of the ISI-*H* correlations, arranged vertically by group, and horizontally by frequency band; electrodes where *p* < 0.05 and *p* < 0.1 (corrected for multiple comparisons after Threshold-Free Cluster Enhancement) are plotted with white and black dots, respectively. Note the more prominent positive correlations between *H* and ISI at low frequencies for ID and at high frequencies for CTRL.

In order to determine whether the association between LRTC and the severity of insomnia complaints had a spectral profile specific to each group, we examined for each frequency band the Spearman correlation coefficients and their confidence intervals, for ID and CTRL. Both in ID and CTRL, the EO *H* exponents correlated positively with ISI in all frequency bands. Robust correlations (consistently positive across more than 97.5% of the bootstrap iterations) were observed in different frequency bands for each group (Figure 4C). In ID the associations were more robust in the low-frequency bands, *rho* (bootstrap 95% C.I.): theta = 0.335 (0.086–0.551); alpha = 0.312 (0.054–0.572); sigma = 0.276 (0.024–0.532); beta-1 = 0.236 (−0.04–0.491); beta-2 = 0.198 (−0.008–0.449). In CTRL the associations were more robust in the high-frequency bands: theta = 0.165 (−0.206–0.466); alpha = 0.102 (−0.266–0.396); sigma = 0.327 (0.049–0.583); beta-1 = 0.426 (0.210–0.674); beta-2 = 0.392 (0.153–0.630).

In order to further detail the association between LRTC and the severity of insomnia complaints at the spatio-spectral level, Spearman correlations were calculated for each frequency-electrode bin. Statistical significance, corrected for multiple comparisons, was assessed with threshold-free cluster enhancement (TFCE). Positive correlations between ISI and *H* in each group were found over extended regions and in multiple frequency bands (Figure 4D). In the following, we report the spatial extent (number of electrodes with *p* < 0.05) and the peak intensity (*rho, t, t*_*tfce*_, and *p* at the electrode with maximal statistical evidence), separately for each frequency band.

**Figure 5:**
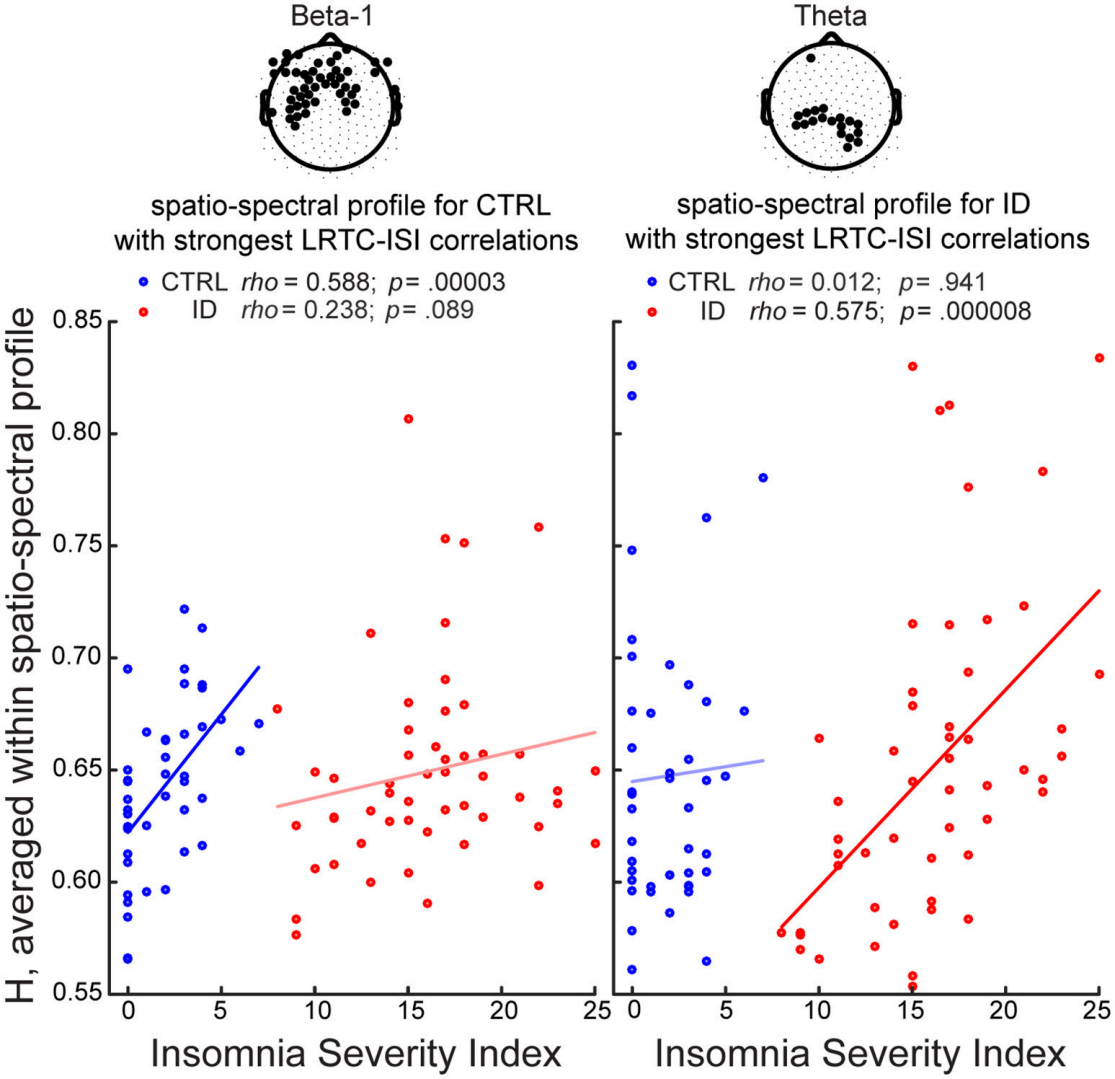
**Long-Range Temporal Correlations in EEG amplitude fluctuations during Eyes Open increase with severity of insomnia complaints, particularly within parietal theta oscillations in participants with Insomnia Disorder, and within frontal beta-1 oscillations in matched controls**. We identified for each group the spatio-spectral profile with the strongest association between *H* and the Insomnia Severity Index (ISI), and visualized the correlations in both groups. The group-specific spatio-spectral profile is defined in two steps: (1) we first selected the frequency band where the largest number of electrodes displayed significant correlations between *H* and ISI (*p* < 0.05, corrected for multiple comparisons after Threshold-Free Cluster Enhancement); (2) we then averaged the *H*-values at that frequency band, across those electrodes (highlighted in black in the topographies). In controls (CTRL), the strongest spatio-spectral correlate was the average across electrodes in a frontal region extending to bilateral central and fronto-temporal regions in the beta-1 band. In people with Insomnia Disorder (ID), the strongest spatio-spectral correlate was the average across parietal electrodes in the theta band. A least-squares line is shown for each group. Within-group Spearman correlation coefficients (*rho*) and their respective *p*-values (uncorrected) are shown on top.

In the ID group, ISI positively correlated with *H*, in a bilateral parietal region within the theta band (18 electrodes; peak *rho* = 0.521, *t* = 4.317, *t*_*tfce*_ = 245.032, *p* = 0.027) (as illustrated further in Figure 5), in a frontal region within the alpha band (7 electrodes; peak *rho* = 0.393, *t* = 3.020, *t*_*tfce*_ = 206.180, *p* = 0.048), in prefrontal and right fronto-temporal regions within the sigma band (10 electrodes; peak *rho* = 0.443, *t* = 3.489, *t*_*tfce*_ = 215.432, *p* = 0.041), and, be it only in small regions, in midline prefrontal and right fronto-temporal regions within the beta-1 band (7 electrodes; peak *rho* = 0.458, *t* = 3.647, *t*_*tfce*_ = 217.597, *p* = 0.038).

In the CTRL group, ISI positively correlated with *H*, in midline prefrontal and left frontal regions within the sigma band (9 electrodes; peak *rho* = 0.468, *t* = 3.390, *t*_*tfce*_ = 197.980, *p* = 0.032), in a frontal region extending to bilateral central and fronto-temporal regions within the beta-1 band (48 electrodes; peak: *rho* = 0.547, *t* = 4.181, *t*_*tfce*_ = 269.731, *p* = 0.012) (as illustrated further in Figure 5), and in bilateral fronto-temporal and left central regions within the beta-2 band (23 electrodes; peak *rho* = 0.568, *t* = 4.414, *t*_*tfce*_ = 276.426, *p* = 0.010).

## Discussion

We investigated whether LRTC in the amplitude fluctuations of band-filtered EEG oscillations during the wake resting state differ between people suffering from Insomnia Disorder and matched controls. We moreover investigated whether individual differences in these autocorrelations are associated with individual differences in the severity of insomnia complaints. For this purpose, we estimated the scaling exponent of LRTC through the Hurst exponent, derived from Detrended Fluctuation Analysis.

The results indicate that people suffering from Insomnia Disorder *grosso modo* do not show different strength of LRTC as compared to controls. However, within each group, individuals experiencing worse sleep quality tend to have stronger LRTC during eyes open wakefulness. Furthermore, the association between insomnia complaints and LRTC has a distinct spatio-spectral profile in each group, suggesting that people with insomnia and matched controls have different neural correlates of subjective insomnia complaints.

Within the physiological dynamical range (Priesemann et al., 2014), higher LRTC are indicative of a higher excitation to inhibition ratio (Poil et al., 2012) (see Figure 1). In the present paper, we speculate that stronger LRTC in people experiencing more severe insomnia complaints reflect increased excitability of cortical networks. This is in agreement with recent reports on decreasing LRTC with sleep depth (Tagliazucchi et al., 2013; Kantelhardt et al., 2015), and with the hypothesis that sleep contributes to the homeostasis between excitatory and inhibitory processes in the brain (Pearlmutter and Houghton, 2009).

### People suffering from ID and controls show similar LRTC

We expected a higher excitation to inhibition ratio in people suffering from ID as compared to matched controls; therefore we hypothesized that they would show stronger LRTC. However, the groups did not differ either at the grand-median level, or at the fine-grained spatio-spectral level (Supplementary Material). One explanation for the lack of group differences is that ID entails an elevated excitation to inhibition ratio beyond the critical point, resulting in similar LRTC to those of matched controls (Poil et al., 2012) (see Figure 1). However, such a scenario seems unlikely, given that the physiological range of brain dynamics throughout different vigilance states, species, recording techniques and comparative simulations stays below the critical point (Priesemann et al., 2013, 2014). Furthermore, in our experiment, the association between LRTC and the severity of insomnia complaints was positive in each group, as predicted assuming that brain dynamics are below the critical point in both groups.

Another possible explanation for the lack of group differences in LRTC is that the neural correlates of subjective insomnia complaints are reflected in dynamical processes that are distinct in each group. We discuss this possibility in the next section.

### The severity of insomnia complaints in ID and controls increases with LRTC during EO

We observed a positive association between LRTC during Eyes Open and the severity of insomnia complaints, in people suffering from ID and in matched controls. Such association was specific to each group. Accordingly, higher correlation coefficients were observed within each group than across groups (Supplementary Material). Furthermore, the correlations between the severity of insomnia complaints and LRTC show spatio-spectral profiles that are group-specific. In our control group without insomnia, individuals with mild insomnia complaints, as compared to those with no insomnia complaints, have stronger LRTC in high frequency band power fluctuations, prominently so over frontal and bilateral centro-frontal regions within the beta-1 band. Conversely, among participants suffering from Insomnia Disorder, individuals with severe insomnia complaints, as compared to those with moderate insomnia complaints, have stronger LRTC in low frequency band power fluctuations, prominently so over parietal regions within the theta band (Figures 4C,D and 5). In other words, the severity of insomnia complaints increases with LRTC during eyes open wakefulness, specifically in low frequency oscillations among people with Insomnia Disorder, and specifically in high frequency oscillations among controls. Therefore, the wake brain dynamical processes underlying the severity of insomnia complaints are likely different between people with Insomnia Disorder and matched controls.

We observed that individuals who experience worse sleep quality show stronger LRTC during Eyes Open but not during Eyes Closed. Closing the eyes increases the mean strength of LRTC—as previously observed (Nikulin and Brismar, 2004, 2005). Stronger LRTC during eyes closed wakefulness may reflect a shift from below towards the critical point and thus an increase in the excitation to inhibition ratio. Crucially, the increase in LRTC becomes progressively smaller when approaching the critical point (Poil et al., 2012) (see Figure 1). Furthermore, closing the eyes increases the between-participants variation of LRTC (Supplementary Figure S1), potentially concealing systematic variation of interest across participants in LRTC. Therefore, the analysis of LRTC during eyes closed wakefulness may be less sensitive to individual differences in brain excitability, than is the case for LRTC during Eyes Open.

### Sleep and LRTC

The main finding of the present study is that, within both groups, more severe insomnia complaints parallel stronger LRTC in the amplitude fluctuations of ongoing EEG oscillations. This suggests that a high excitation to inhibition ratio in neuronal networks during wakefulness is linked, in individuals with ID and in controls, to poor sleep quality. Consistently, other studies have found that after falling asleep there is a progressive decrease of LRTC with deeper sleep stages, in both amplitude and frequency fluctuations of EEG oscillations (Kantelhardt et al., 2015), as well as in the amplitude fluctuations of the blood oxygenated level dependent (BOLD) signal in the attention and default mode networks (Tagliazucchi et al., 2013). We speculate that poor sleep quality may insufficiently reduce LRTC during subsequent wakefulness, by insufficiently reducing the excitation to inhibition ratio in neuronal networks. This interpretation is consistent with the hypothesis that sleep plays a key role in keeping the wake brain sufficiently far from dynamics dominated by excitation, to provide a safe margin from uncontrolled runaway activity (Pearlmutter and Houghton, 2009)

### Limitations

The measurement of insomnia severity was based on subjective complaints. Future studies could complement the present study by the use of polysomnography, to evaluate the association of LRTC in resting-state brain dynamics with objective sleep quality, as well as the associations of LRTC in sleep brain dynamics with objective and subjective sleep quality. Future studies may also evaluate LRTC in frequency fluctuations, rather than in amplitude fluctuations, of EEG oscillations (Kantelhardt et al., 2015) in people with insomnia.

### Conclusions

We estimated LRTC in band-filtered amplitude fluctuations of HD-EEG, among people suffering from Insomnia Disorder and matched controls. LRTC were similar across groups. Within each group, people with more severe insomnia complaints have stronger LRTC, during eyes open wakefulness. Furthermore, the association has a spatio-spectral profile specific to each group, suggesting that insomnia complaints are reflected in wake brain dynamical processes that are distinct in people with Insomnia Disorder and controls. Our findings of stronger LRTC with increased severity of insomnia complaints may reflect an increase of brain excitability, suggesting a disruption of the sleep-dependent homeostasis of the excitation-inhibition balance. In a broader perspective, these findings challenge the notion that higher complexity is a signature of better health, and that disorders are associated to a loss of complex dynamics (Yang and Tsai, 2013). Instead, sleep may reduce the brain excitability, yielding intermediate levels of dynamical complexity during wakefulness, in order to prevent the insurgence of seizure-like dynamics.

## Author contribution

MC, YW, JR contributed to data collection; MC, YW, ET performed the analysis; MC wrote the manuscript; JR set up the laboratory for data collection; KL-H, ET provided fruitful interpretation of the data; KL-H, ET, EV oversaw the project; EV designed the data acquisition protocol; All authors participated in the revision of the manuscript.

## Funding

This research was supported by: NeuroTime grant: 520124-1-2011-1-FR-ERA; The Bial Foundation grant 252/12; The AXA Research Fund Junior Postdoctoctoral Research Fellowship 15-AXA-PDOC-150; The Netherlands Organization of Scientific Research (NWO) grant VICI-453.07.001; The European Research Council Advanced grant ERC-ADG-2014-671084 INSOMNIA.

### Conflict of interest statement

The authors declare that the research was conducted in the absence of any commercial or financial relationships that could be construed as a potential conflict of interest.
