# Supplementary material for: More Severe Insomnia Complaints in People with Stronger Long-Range Temporal Correlations in Wake Resting-State EEG
Source: Front Physiol. 2016 Nov 29;7:576. doi: 10.3389/fphys.2016.00576 (PMC5126110; doi:10.3389/fphys.2016.00576)
Supplement: Supplementary file 1 [file DataSheet1.docx]

Supplementary Material

**More Severe Insomnia Complaints in People with Stronger
 Long-Range Temporal Correlations in Wake Resting-State EEG**

**Michele A. Colombo*, Yishul Wei, Jennifer R. Ramautar, Klaus Linkenkaer-Hansen, Enzo Tagliazucchi, Eus J.W. Van Someren**

*** Correspondence:** Michele Colombo, Netherlands Institute for Neuroscience (NIN), Meibergdreef 47, 1105 BA Amsterdam, The Netherlands, +31 (0) 20 566 11 42: m.colombo@nin.knaw.nl

# Differences in LRTC between Eyes Open and Eyes Closed

We compared the two resting-state conditions (Eyes Open and Closed: EO, EC) in terms of the strength of Long-Range Temporal Correlations (LRTC) in the amplitude fluctuations of each frequency band (theta, alpha, sigma, beta-1, beta-2), aggregated across electrodes. For this purpose, the median *H* across electrodes was obtained in each condition. Then a signed-rank test was performed for the within-participants difference (EC – EO). The median *H* across electrodes was larger in EC than in EO, significantly for all bands, except only at trend-level for theta: theta (*z* = 1.871, *p* = 0.061), alpha (*z* = 3.103, *p* = 0.0019), sigma (*z* = 3.519, *p* = 0.0004), beta-1 (*z* = 3.578, *p* = 0.0003), beta-2 (*z* = 4.005, *p* = 0.0001) (Supplementary Figure S1A). As argued in the main manuscript, stronger LRTC during eyes closed wakefulness can index a shift from below towards the critical point, possibly resulting from an increase in brain excitability (see Figure 1 of the main manuscript). Because the increase in LRTC becomes progressively smaller when approaching the critical point (Poil et al., 2012), individual differences in brain excitability might be more concealed under the EC than the EO condition.
Furthermore, we compared the two resting state conditions in terms of the between-participants variation of LRTC in the amplitude fluctuations of each frequency band. Variation was quantified as the median absolute deviation of *H* across participants, at each electrode and frequency band. We then tested for each frequency band whether such variation was larger—consistently across electrodes—in one resting-state condition with respect to the other, by performing a signed-rank test across electrodes on the difference in variation between the two conditions (EC – EO). The between-participants variation of *H* was significantly larger across electrodes in EC than in EO in all bands except for theta: theta (*z* = 0.672, *p* = 0.502), alpha (*z* = 3.399, *p* = 0.0007), sigma (*z* = 5.098, *p* < 0.0001), beta-1 (*z* = 4.447, *p* < 0.0001), beta-2 (*z* = 4.054, *p* = 0.0001) (Supplementary Figure S1B). Larger between-participants variation of LRTC might conceal systematic variation of interest across participants in LRTC.
We conclude that the larger mean and larger between-participants variation of LRTC in EC with respect to EO could have concealed individual differences in LRTC accounted by the Insomnia Severity Index (ISI).

# During EO, the association of the grand-median *H* with ISI is present within each group

We aimed to clarify whether the observed association between the severity of insomnia complaints and the LRTC scaling exponents, aggregated across frequencies and electrodes, held more strongly within each group or across groups. Therefore, we performed two supplementary analyses.
1) For the EO condition, we assessed whether including the group factor significantly improved the general linear model (GLM), by contrasting a GLM that included both ISI and group as predictors to a simpler model with only ISI as the predictor. An ANOVA contrasting the two GLMs revealed that the model including both group and ISI was better than the model with ISI alone (*F* (1,91) = 5.404, *p* = 0.022), suggesting that group was an important factor when considering the association between ISI and grand-median *H*, aggregated across frequencies and electrodes.
2) Spearman-correlations between ISI and the grand-median *H* were performed both irrespective of groups (ALL), and within each group (in ID and in CTRL), for the EO as well as the EC condition.
In EO, the effect of ISI was significant in both ID and CTRL, while it only showed a trend in ALL (Supplementary Figure S2, left). In EC, correlations were not significant: The effect of ISI showed a trend in ID, but not in CTRL, and a trend in ALL (Supplementary Figure S2, right). The lack of associations during EC is consistent with the results seen from the univariate GLM during EC (see the main manuscript, in the Result section “*During EO, the Grand-Median Hurst Exponent Increases with ISI, in ID and in CTRL*”). The results obtained during EO suggest that there is an association between LRTC and ISI that holds stronger within each group, rather than across groups. Accordingly with this argument, such association had a spatio-spectral profile that was specific for each group, as detailed in the main manuscript, in the Result section “*Group-Specific Spectral and Spatio-Spectral Profiles of Correlations between ISI and H during EO*”.

# LRTC do no differ between ID and CTRL

During the EO and EC resting states, we did not observe any between-group differences in LRTC, aggregated across frequencies and electrodes, as indicated by Wilcoxon rank-sum tests on the grand-median *H*. However, a local effect at specific electrodes and frequencies could be masked when the grand-median is taken. In order to observe whether insomniacs differed from matched controls with respect to LRTC with a specific spatio-spectral profile, we conducted a Wilcoxon rank-sum test with respect to *H*, at each electrode and frequency. The tests were performed only for the EO condition, where group was found to be a significant factor, together with ISI, in explaining variation of the grand-median *H*, according to the GLM. The *z*-statistics were enhanced, following the threshold-free cluster enhancement (TFCE) procedure, yielding *z_tfce_* values. We then assessed the significance of each enhanced statistic by comparing it to the empirical null hypothesis distribution, constructed by Monte Carlo permutation with 1000 iterations. This procedure was analogous to the one followed for the within-group spatio-spectral correlations (see Methods section of the main manuscript).
The effect of group was statistically non-significant at all frequencies and electrodes. The maximal evidence obtained was in beta-1 at electrode #182, with *z* = 2.666, *z_tfce_ =* 36.197, *p* = 0.693. We conclude that insomniacs did not exhibit a larger amount of LRTC in any spatio-spectral range during the wake resting state.

# Age and Sex are matched between groups and are not associated to ISI

We compared insomniacs to matched controls with respect to age and sex. We then estimated whether ISI was associated with age, or whether it was different for males and females. Wilcoxon rank-sum tests revealed that the two groups did not differ with respect to age (*z* = 1.290, *p* = 0.197), or sex (*z* = -.974, *p* = 0.330). ISI did not correlate with age, as revealed by Spearman correlations performed irrespective of group (*t*(92) = 0.727, *p* = 0.469), within the ID group (*t*(50) = -1.422, *p* = .161), or within the CTRL group (*t*(41) = 0.347, *p* = 0.730). Furthermore, ISI did not differ between sexes (*z* = -.344, *p* = 0.731).

# Supplementary Figures


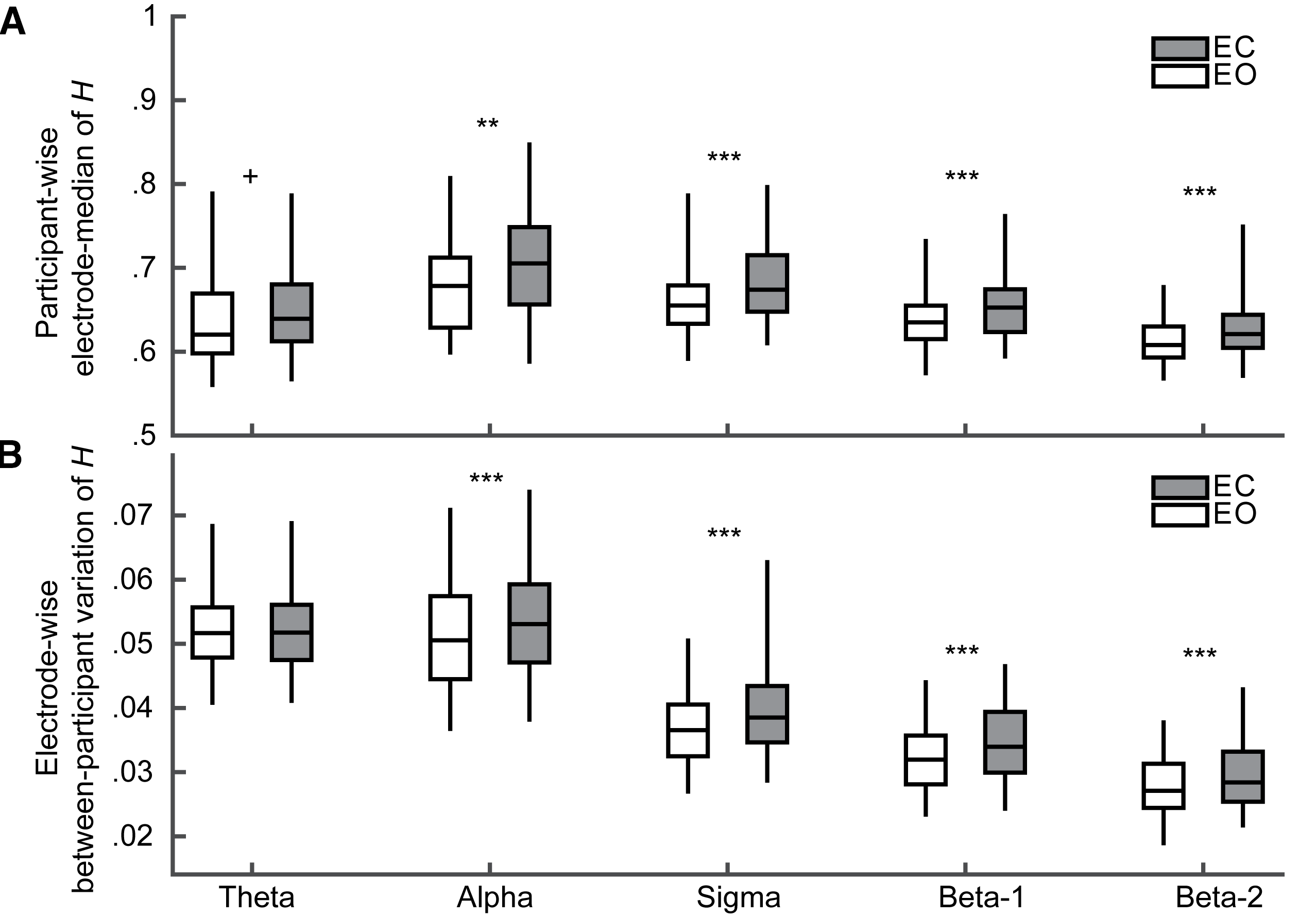


**Supplementary Figure S1. The median *H* across electrodes and the between-participants variation of *H* was larger in Eyes Closed (EC) than in Eyes Open (EO).** (**A**): The median *H* across electrodes was larger in EC than in EO, significantly so for alpha, sigma, beta-1 and beta-2, and at a trend-level in theta. (**B**): The between-participants variation of *H* was larger, across electrodes, in EC than in EO, significantly so for alpha, sigma, beta-1 and beta-2. Symbols code the *p*-values: + for *p* < .1, * for *p* < .05, ** for *p* < .01, *** for *p* < .001. The variation is computed at each electrode as the median absolute deviation of *H* across all participants.

**
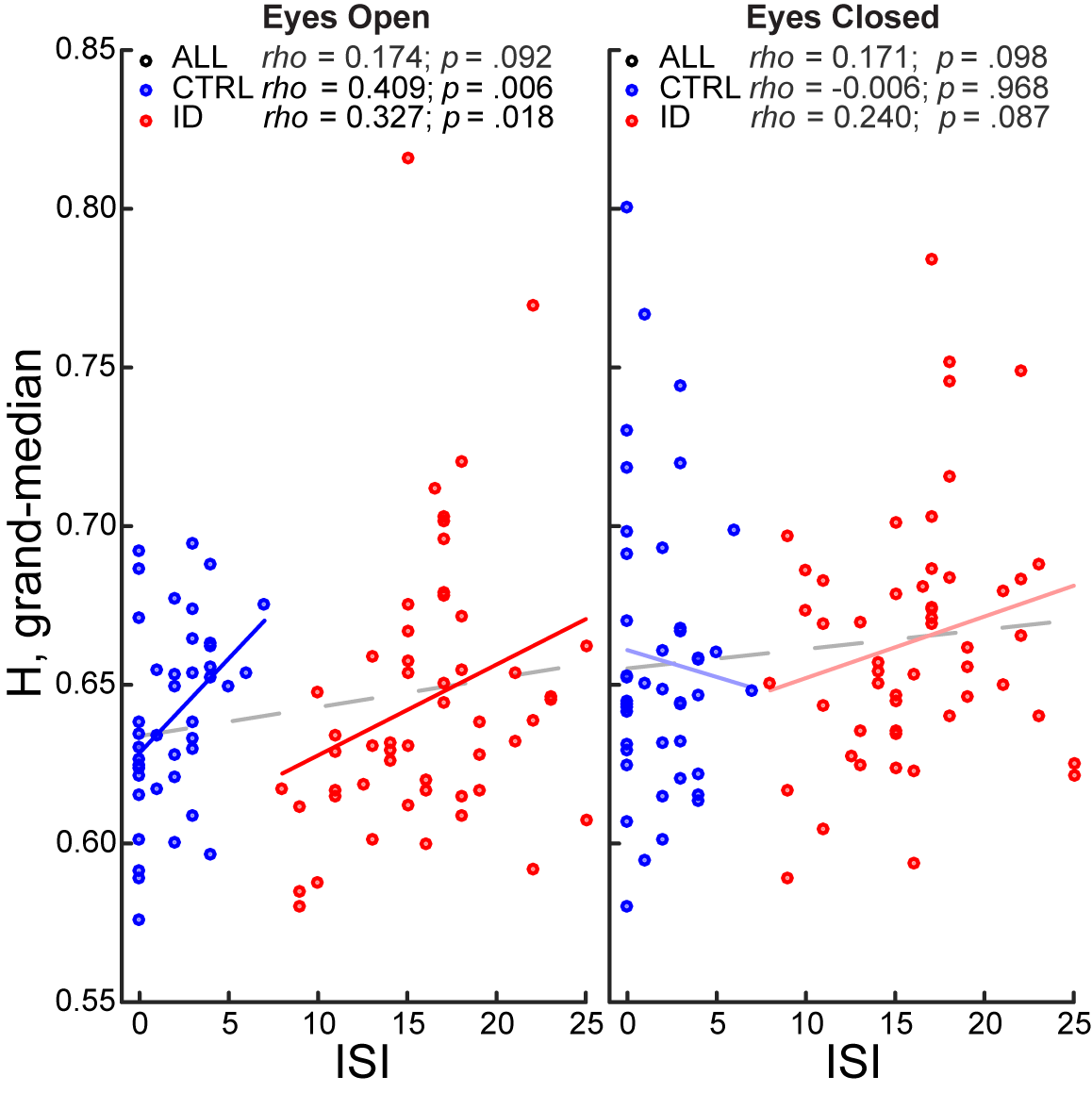
**

**Supplementary Figure S2. During Eyes Open, but not during Eyes Closed, the LRTC scaling exponent—aggregated across frequencies and electrodes— of EEG amplitude fluctuations positively correlates with the Insomnia Severity Index (ISI), in participants with Insomnia Disorder (ID) and in matched controls (CTRL**). During Eyes Open the correlation was significant within each group (in ID and in CTRL), while it only displayed a trend across all participants (ALL). During Eyes Closed the correlation displayed a trend in ID and in ALL, while no correlation was present in CTRL. A least square line is shown for each group. Spearman correlation coefficients and their respective *p*-values are shown on top. This graph complements the results of the GLM, described in the Result section of the main manuscript “*During EO, the Grand-Median Hurst Exponent Increases with ISI, in ID and in CTRL*”.
